# Preliminary Study on Phonation Reconstruction Using Free Anterolateral Thigh and Sternohyoid Myocutaneous Flaps After Total Laryngectomy

**DOI:** 10.1002/cam4.71294

**Published:** 2025-10-08

**Authors:** Hongming Wei, Jian Li, Huilei Dong, Yang Xu, Xin Li, Zhu Liu, Tie Lu, Bin Li, Zhendong Li, Hongwei Liu

**Affiliations:** ^1^ Liaoning Cancer Hospital & Institute Shenyang China; ^2^ The People's Hospital of Yingkou Yingkou China

**Keywords:** free anterolateral thigh flap, perioperative nutrition, phonation reconstruction, postoperative care, sternohyoid myocutaneous flap, total laryngectomy

## Abstract

**Objective:**

In this study, we evaluated and compared the outcomes of phonation reconstruction using free anterolateral thigh (ALT) and sternohyoid myocutaneous flaps in patients undergoing total laryngectomy for locally advanced laryngeal and hypopharyngeal cancers, providing insights into optimal reconstructive approaches.

**Materials and Methods:**

A total of 10 patients with locally advanced laryngeal or hypopharyngeal at Liaoning Cancer Hospital & Institute were enrolled. All patients underwent total laryngectomy with simultaneous voice reconstruction. The reconstruction method was selected based on the extent of the hypopharyngeal defect. For patients without mucosal defects in the hypopharynx (*n* = 7), a sternohyoid myocutaneous flap was used. For those with large mucosal defects (*n* = 3), a free ALT flap was applied for voice reconstruction. Quality of life (QoL) was assessed preoperatively and postoperatively using a modified Chinese version of the University of Washington Quality of Life (UW‐QOL) questionnaire. Postoperative complications were analyzed.

**Results:**

Both ALT and sternohyoid myocutaneous flaps demonstrated good feasibility and safety in phonation reconstruction following total laryngectomy. Phonation was achieved intraoperatively without the need for additional voice prosthetic devices. No statistically significant differences were observed between the groups regarding QoL or postoperative complications.

**Conclusion:**

Preliminary findings suggest that both ALT and sternohyoid myocutaneous flaps are viable options for phonation reconstruction following total laryngectomy. Phonation reconstruction contributed to a moderate improvement in patients' quality of life post‐laryngectomy. Strengthening perioperative management is essential for optimizing surgical outcomes. Further studies with larger sample sizes and controlled designs are warranted to validate these findings and provide more robust evidence to guide the selection of surgical techniques in clinical practice.

## 1 Introduction

Laryngeal and hypopharyngeal cancers are among the most common malignant tumors of the head and neck [1], and total laryngectomy (TL) is the primary treatment for locally advanced cases. As the larynx is crucial for phonation, losing this function significantly impacts the quality of life of patients, resulting in substantial physical and psychological burdens. Surgical voice rehabilitation offers functional recovery, significantly improving quality of life post‐laryngectomy [2]. Consequently, voice rehabilitation has become integral to treatment for laryngeal or hypopharyngeal cancers, given that the motor for phonation and articulation organs [3]. After Theodor Billroth performed the first TL in 1873, Carl Gussenbauer developed a mechanical device in 1874 to compensate for voice loss, representing the earliest documented attempt at artificial laryngeal reconstruction in the context of TL [4, 5].

Modern phonation reconstruction methods post‐TL fall into three categories: esophageal speech, artificial larynx, and tracheoesophageal speech [6]. Esophageal speech, the earliest voice reconstruction method, involves storing air in the esophagus, which is expelled to produce weak sounds later shaped into speech by the tongue, lips, and teeth [7]. Clinical reports indicate a 70%–88% success rate, with benefits such as no need for external devices or secondary surgery and a relatively natural tone. However, it requires prolonged training, produces lower sound frequencies, lacks the coherence of electronically generated sounds. Furthermore, approximately 30% of patients are unable to master this technique.

The artificial larynx is commonly used in clinical practice and is categorized into electronic and mechanical devices. The electronic larynx generates sound through electronic oscillations and electromagnetic vibrations, which are articulated into speech by the pharynx and oral cavity. Its advantages include a high success rate (up to 91.3%) and ease of use, requiring minimal training [8]. However, the drawbacks include unnatural intonation, background noise, and the need for handheld operation, which may disrupt daily activities [9]. The mechanical artificial larynx uses a funnel‐shaped device placed over the tracheostoma, where airflow vibrates a membrane, transmitting sound to the pharynx for articulation. It is user‐friendly and produces relatively clear speech but requires frequent cleaning and is often socially inconvenient due to its external nature. Its monotonous sound quality and unsuitability for patients with oral or tongue impairments are additional limitations [10].

Tracheoesophageal speech (TES), a surgical approach, has become a significant focus in post‐TL phonation reconstruction. Tracheoesophageal puncture (TEP) is particularly effective, with over 75% of patients reporting significant quality‐of‐life improvements [11]. TES methods include non‐prosthetic and prosthetic techniques. Non‐prosthetic TES involves surgically creating a fistula between the trachea and esophagus to allow lung airflow for sound production, later articulated into speech. Arslan et al. pioneered this technique in 1972, but complications such as difficult decannulation and aspiration are evident, and its indications remain limited [12]. Prosthetic TES, involving voice prostheses, is more common due to improved outcomes and fewer complications. Nevertheless, phonation valves typically require replacement every 3 months and are relatively expensive, with patients always having to carry them. The cleaning instruments must be used daily, and the maintenance process is cumbersome. In addition, there is a tendency for granulation tissue formation, valve dislodgement, and even tracheal foreign body formation due to accidental aspiration of the valve.

With technological advancement over time, external artificial larynx devices have continued to evolve and improve; however, they still present certain limitations as external apparatuses. Therefore, researchers have continuously been striving to improve methods of voice reconstruction after TL. Continued innovations in phonation reconstruction methods, including the use of free ALT and sternohyoid myocutaneous flaps, reflect ongoing efforts to improve outcomes for patients undergoing TL. Both the free ALT flap and the sternohyoid myocutaneous flaps can be harvested autologously and allow for synchronous surgical repair and reconstruction. Patients can easily practice phonation postoperatively by covering the tracheal stoma with the thumb or a simple stoma valve. This enables airflow entering through the phonation tube via its external opening and exiting through the internal opening, directing airflow to the esophageal or pharyngeal mucosa, where sound is generated by the articulatory organs. Furthermore, both flap harvesting and reconstruction procedures are technically mature, with a success rate exceeding 95%. Once patients exhibit good recovery, phonation function can be stably maintained for years without the need for prosthetic voice valves or electrolarynx devices, as required in other phonation methods. Moreover, complications are relatively rare and manageable, significantly reducing the financial burden and psychological stress for patients [13]. These advancements provide a foundation for enhancing post‐laryngectomy quality of life through effective and sustainable voice rehabilitation. In this study, we aimed to investigate the effectiveness of using free ALT flap and sternohyoid myocutaneous flap for phonation reconstruction following TL and to preliminarily summarize the advantages, disadvantages, and feasibility of these two reconstructive approaches.

## 2 Materials and Methods

### 2.1 Study Subjects

A total of 10 patients with locally advanced laryngeal (*n* = 7) or hypopharyngeal (*n* = 3) cancer at Liaoning Cancer Hospital & Institute (9 males and 1 female; aged 51 to 68 years, mean 60.6 years) were enrolled in this study. All patients underwent total laryngectomy with simultaneous voice reconstruction. The treatment protocol was as follows: voice reconstruction was performed following total laryngectomy. The inclusion criteria were patients who underwent total laryngectomy, had normal finger dexterity, aged < 75 years, had good pulmonary function, and had a desire to speak. The exclusion criteria were poor cardiopulmonary function, impaired finger mobility, or a history of preoperative radiotherapy. The patients' baseline characteristics and TNM staging information are presented in Table 1.

**TABLE 1:** Baseline characteristics of the patients in this study.

Patient No.	1	2	3	4	5	6	7	8	9	10
Sex	Male	Male	Male	Male	Male	Male	Male	Male	Female	Male
Age (years)	53	67	51	62	67	68	67	61	59	59
Diagnosis	Laryngeal	Hypopharyngeal	Hypopharyngeal	Laryngeal	Laryngeal	Laryngeal	Laryngeal	Laryngeal	Laryngeal	Hypopharyngeal
TNM Stage	T2N3M0, Stage IV	T3N2M0, Stage IV	T3N0M0, Stage III	T3N0M0, Stage III	T3N2M0, Stage IV	T2N1M0, Stage III	T1N1M0, Stage III	T3N0M0, Stage III	T3N0M0, Stage III	T3N3M0, Stage IV
Smoking history (> 20 pack‐years)	YES	YES	YES	YES	YES	YES	YES	YES	YES	YES
History of prior radiotherapy	NO	NO	NO	NO	NO	NO	NO	NO	NO	NO
Preoperative albumin level	Normal	Normal	Normal	Normal	Normal	Normal	Normal	Normal	Normal	Normal

### 2.2 Flap Selection Criteria

The choice of the reconstruction method should be based on a comprehensive assessment of the patient's specific clinical characteristics. For cases with large mucosal defects following resection of the primary tumor, a free ALT flap—providing ample tissue volume—is preferred. In contrast, for patients with no or minimal mucosal defects, the thinner and more pliable sternohyoid myocutaneous flap is considered more suitable. In this study, phonation reconstruction using a free ALT flap was performed in 3 patients (2 with hypopharyngeal cancer and 1 with laryngeal cancer). In the remaining 7 patients, the sternohyoid myocutaneous flap was utilized for phonation reconstruction (1 with hypopharyngeal cancer and 6 with laryngeal cancer). All the surgeries were performed by the same surgeon and surgical team.

### 2.3 Statistical Methods

Categorical data are expressed as frequencies (n) and percentages (%), and the chi‐square (χ^2^) test was used to compare differences between groups. Continuous data are presented as mean ± standard deviation (x̄ ± s), and the *t* test was used for intergroup comparisons. A *p*‐value < 0.05 was considered statistically significant.

### 2.4 Phonation Reconstruction With Anterolateral Thigh Flap

#### 2.4.1 Surgical Procedure

Following the standard neck lymph node dissection, a tracheostomy was performed at the 4th and 5th tracheal cartilage rings. The trachea was transected at the first tracheal ring, maintaining a 2 cm safety margin. TL or TL with partial hypopharyngectomy was subsequently performed. The flap design varied depending on the extent: for TL alone, it catered solely to phonation tube requirements, whereas for TL with partial hypopharyngectomy, it addressed both the phonation tube and hypopharyngeal defect repair.

#### 2.4.2 Flap Design and Preparation

**FIGURE 1:**
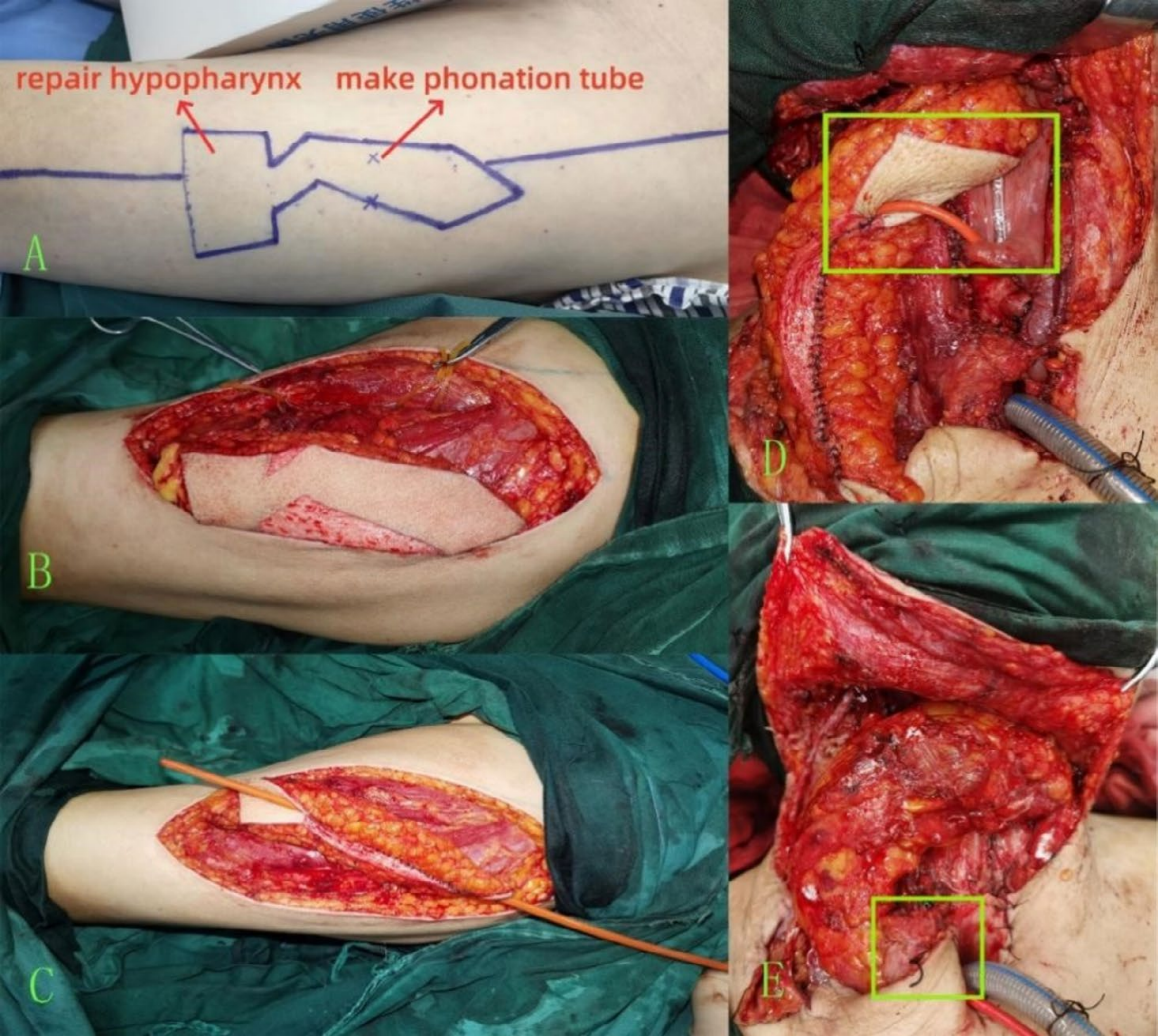
(A) Design of the anterolateral thigh flap prior to preparation. (B) Preparation of the anterolateral thigh flap and removal of excess skin. (C) Shaping of the phonation tube. (D) Repair of the hypopharyngeal defect. (E) Suturing the lower end of the phonation tube.

For TL with partial hypopharyngectomy, the free ALT flap was configured into J‐shaped and rectangular sections. The rectangular section was used to repair the hypopharyngeal defect, whereas the J‐shaped portion was used to form a phonation tube designed with an upward bend at the corner to minimise aspiration. The flap was harvested based on the design (Figure 1A), with excess skin excised (Figure 1B). The phonation tube was shaped in a cylindrical structure using a 16‐gauge Foley catheter as a mould (Figure 1C). Post‐dissection and laryngectomy, the prepared flap was placed on the neck: the rectangular portion was sutured to the reconstructed hypopharyngeal cavity (Figure 1D), and the lower end of the phonation tube was attached to the tracheostomy sidewall (Figure 1E).

#### 2.4.3 Important Considerations for Preparing the Anterolateral Thigh Flap

**FIGURE 2:**
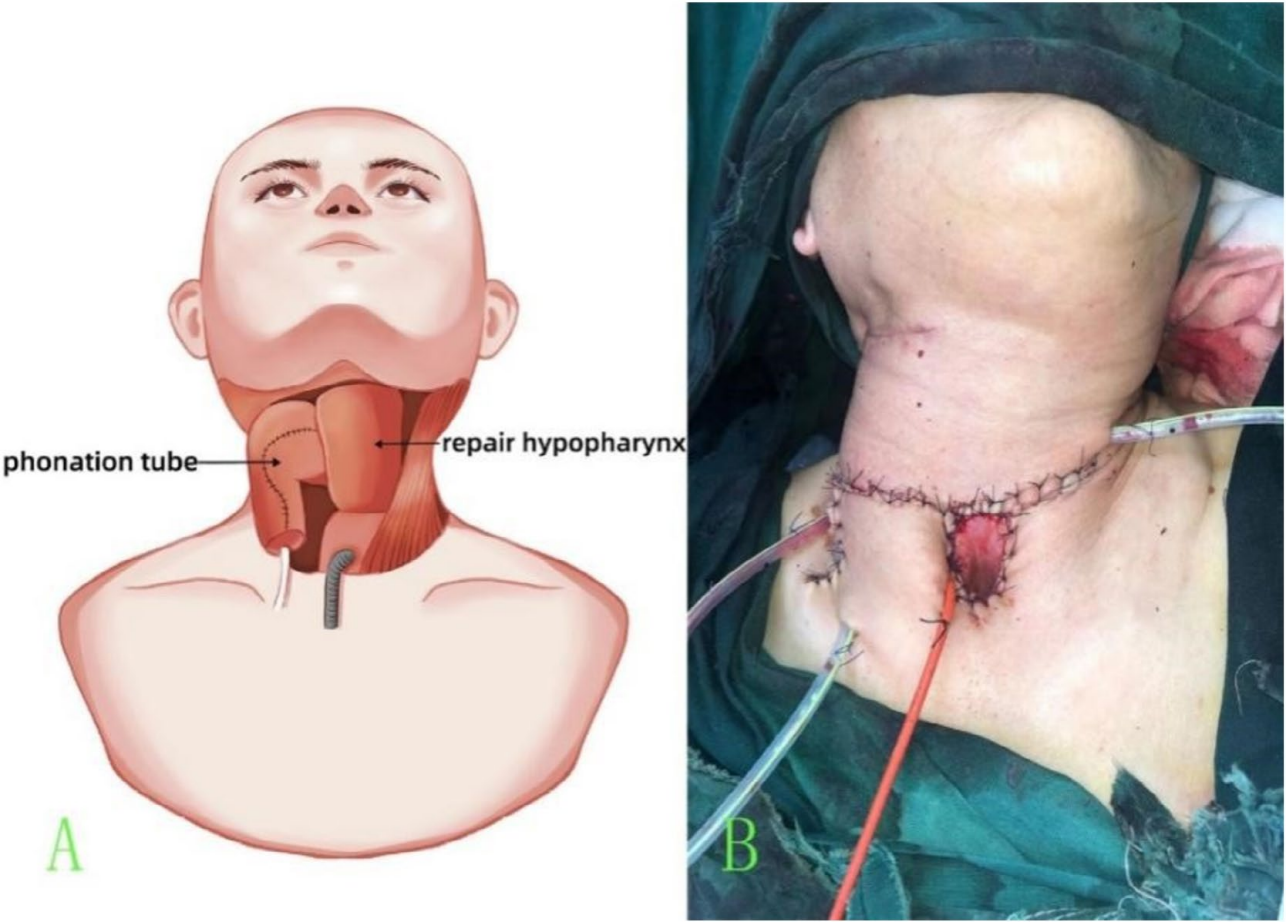
(A) Reconstruction model for hypopharyngeal repair and phonation tubes (B) Completed repair of the neck surgical area.

The size of the rectangular flap was determined by the hypopharyngeal defect dimensions. The length and width of the flap section used for the phonation tube depended on the defect length at the tracheal stump and the diameter of the tube. The curvature angle of the tube, crucial for preventing aspiration, was adjusted to its length; shorter tubes required smaller angles, with a greater risk of aspiration. A 16‐gauge Foley catheter was inserted into the phonation tube to prevent adhesion. The reconstruction model for hypopharyngeal repair and phonation aimed to restore both structural integrity and functional phonation (Figure 2A). The final neck repair outcome appeared optimal (Figure 2B).

### 2.5 Sternohyoid Myocutaneous Flap for Phonation Reconstruction

**FIGURE 3:**
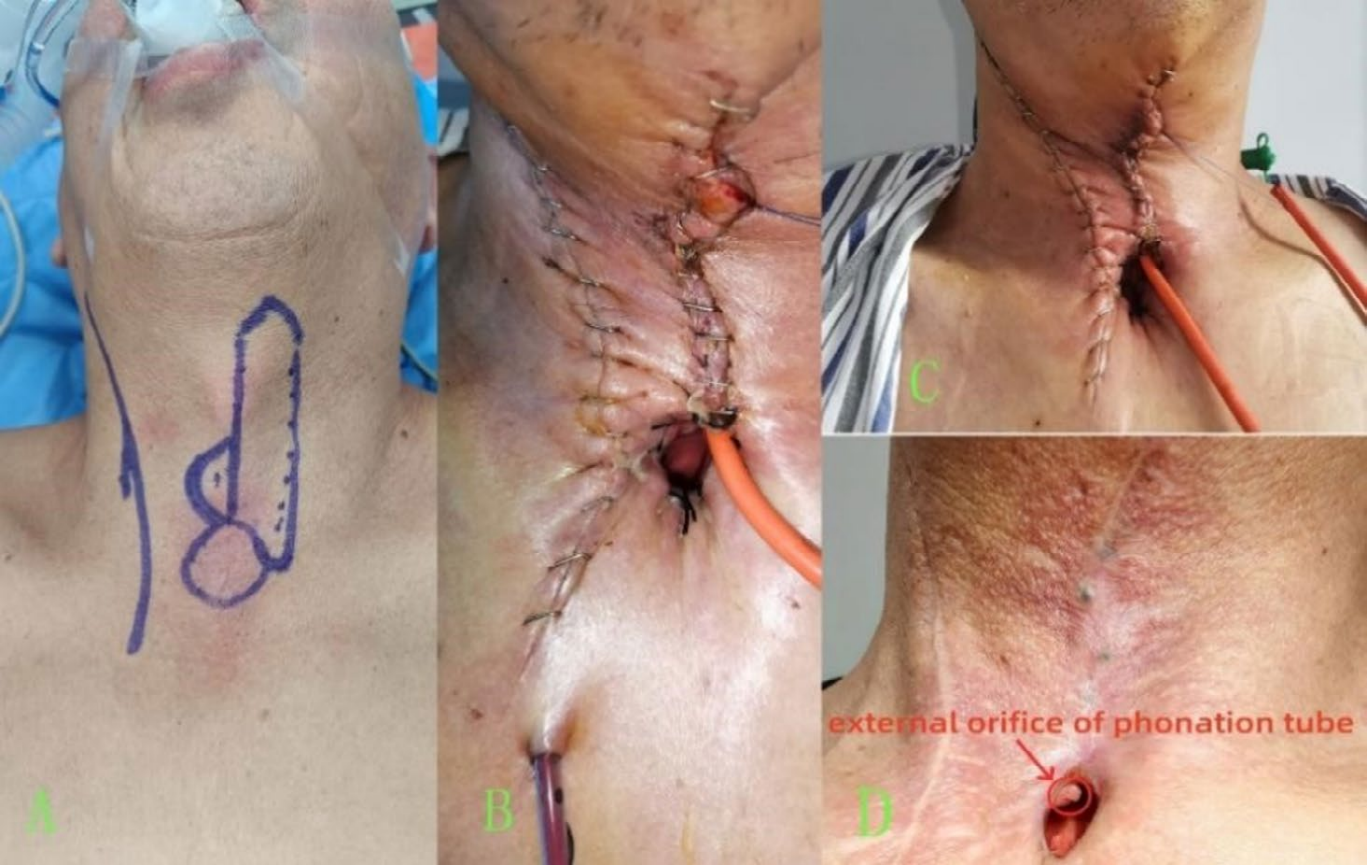
(A) Design of the sternohyoid myocutaneous flap prior to preparation. (B) Good alignment of the suture site on postoperative Day 1. (C) Neck recovery status 1 week post‐surgery. (D) Neck recovery status 1 month post‐surgery.

A sternohyoid myocutaneous flap was designed before preparation (Figure 3A). The circle marked the planned tracheostomy site, while the semicircle indicated the contralateral sternohyoid myocutaneous flap, which was used to form the phonation tube. The skin was incised along a vertical line down to the deep surface of the sternohyoid muscle. The flap was harvested, preserving its blood supply, for phonation tube construction. The width of the local flap was carefully tailored based on the required diameter of the phonation tube, which varied according to individual patient anatomy and reconstruction needs. Generally, a narrower flap ensures better vascularization, which is crucial for flap viability and healing. Therefore, the flap design was individualized to balance adequate lumen size for phonation with optimal perfusion for minimizing complications.

Following TL, the pharyngeal cavity was reconstructed and sutured, leaving a 5 mm opening. The sternohyoid myocutaneous flap was wrapped around a 16‐gauge Foley catheter to form a tubular structure. The external opening of the tube was aligned with the tracheostomy site and sutured, while the internal opening was attached to the reserved pharyngeal cavity opening. The phonation tube comprised the sternohyoid myocutaneous flap, a small contralateral skin portion, and the anterior esophageal mucosa wall. The remaining anterior neck skin flap was repositioned and sutured.

#### 2.5.1 Precautions for Preparing Sternohyoid Myocutaneous Flaps

The flap width was carefully determined using minimal contralateral skin and anterior esophageal mucosa to avoid complications. Overly wide flaps may result in necrosis, while narrow flaps could hinder proper phonation tube closure. The sternohyoid myocutaneous flap was sutured into a tubular structure with a 16‐gauge catheter inside to prevent adhesion formation.

### 2.6 Scientific and Well‐Structured Perioperative Training

In this study, training began when conditions permitted: no infection, normal swallowing, no aspiration during eating or drinking, and an adequate physical and mental state.

Preoperative diaphragmatic breathing exercises—deep inhalation followed by slow exhalation lasting twice as long—are essential. The training occurred in a quiet environment, encouraging adequate breathing, emotional stability, and gradual practice of multi‐syllabic and continuous phonation. Educational materials, including text, pictures, recordings, and videos, were used to prepare patients and families for training.

Patients began practicing oral intake of water upon demonstration of good healing of the surgical site after 2 weeks. Once free of aspiration or fistula, the catheter was removed, and speech guidance began. The training involved occlusion phonation, relaxing the body and neck, a slight head tilt, and directing pulmonary airflow through the phonation tube. Patients modulated airflow using the tongue, palate, cheeks, nasal cavity, teeth, and lips to produce sound. Postoperative training progressed from counting to phrases, sentences, and conversations, gradually increasing frequency. This enabled coordination of breathing and phonation, reducing air leakage and enabling normal communication.

### 2.7 Quality of Life Assessment

This study employed a modified Chinese version of the University of Washington Quality of Life (UW‐QOL) questionnaire to assess patient quality of life preoperatively and 3 months postoperatively. The questionnaire focuses on eight aspects: speech, swallowing, appearance, pain, taste, saliva, mood, and recreation. Each scores up to 12.5 points, with one point deducted for each progressive decline in function. For example, in the speech aspect: A. My speech is the same as before—12.5 points; B. I have some difficulty with certain words, but I can still be understood over the telephone—11.5 points; C. Only my family and friends can understand me—10.5 points; D. No one can understand my speech—9.5 points. The scoring in the remaining aspects follows the same principle, totaling 100 points; higher scores indicate better quality of life. Differences in complications, including flap necrosis, pharyngeal fistula, and aspiration with choking/coughing, were also compared between the two surgical methods.

## 3 Results

### 3.1 Patient Characteristics

A total of 10 patients who underwent phonation reconstruction following TL was included in this study. Among them, 3 patients received phonation reconstruction using a free ALT flap, while 7 underwent reconstruction with a sternohyoid myocutaneous flap. None of the patients had a history of preoperative radiotherapy. All patients were in good nutritional and mental state and demonstrated a strong desire to restore speech. Postoperatively, all patients participated in standardized rehabilitation training. The detailed baseline characteristics of the patients, including age, sex, primary diagnosis, TNM stage, smoking history, history of prior radiotherapy, and preoperative albumin levels, are summarized in Table 1

### 3.2 Technical Feasibility

All patients successfully underwent phonation reconstruction without intraoperative failure and recovered well postoperatively. Patients began practicing oral intake of water upon demonstration of good healing of the surgical site after 2 weeks. Once free of aspiration or fistula, the catheter was removed, and speech guidance began. Patients retained articulation organs, enabling effective training despite a changed sound source. These findings demonstrate that both the ALT and the sternohyoid myocutaneous flaps are clinically feasible and safe options for phonation reconstruction.

### 3.3 Functional Outcomes

#### 3.3.1 Speech Function

All patients completed postoperative speech training and gradually progressed from counting to phrases, sentences, and engaging in daily conversation. Three months postoperatively, all patients achieved clearer, more coherent speech. Vocal quality matched preoperative levels, with sufficient loudness, excellent intelligibility, and a strong desire for verbal communication.

#### 3.3.2 Swallowing Function

The nasogastric tube was retained for 15, 13, 16, 15, 14, 15, 14, 13, 15, and 13 days in the 10 patients, with a mean duration of 14.3 days. If the surgical site demonstrated good healing, patients could begin practicing oral intake of water, followed by a gradual transition from soft food to semi‐liquid and liquid diets, progressively advancing to a normal diet. In each group, one patient experienced mild aspiration within an acceptable range, which was managed successfully, indicating satisfactory postoperative recovery of swallowing function in both groups.

#### 3.3.3 Phonation Tube Morphology and Aspiration Findings

Laryngoscopy revealed a patent phonation opening in the hypopharynx, and neck computed tomography confirmed the presence of the phonation tube. The sternohyoid flap phonation tube measured approximately 6–7 cm. Mild aspiration during drinking was observed and was manageable with light external neck pressure (light pressure on the phonation tube). For patients who underwent free ALT flap reconstruction, the phonation tube assumed a curved ‘sickle‐shaped’ configuration after flap placement. For patients who underwent sternohyoid myocutaneous flap reconstruction, the phonation tube showed a smaller calibre. No external neck pressure was required to assist in preventing aspiration.

### 3.4 Postoperative Complications

The main postoperative complications observed in both groups, including necrosis, pharyngocutaneous fistula, and aspiration with choking/coughing, are summarized in Table 2. Each group had one acceptable aspiration case, which was managed by providing small, frequent meals and adjusting head positions. In the sternohyoid group, partial necrosis around the external stoma occurred due to excessive muscle dissection but healed within 2 weeks of debridement and enhanced wound care. The necrosis, fistula, and aspiration rates for the two flaps were 0% and 14.3%; 33.3% and 14.3%; 33.3% and 14.3%, respectively. No statistically significant differences in necrosis, fistula, or aspiration rates were observed between the groups. The risk factors for pharyngocutaneous fistula in patients included in this study are shown in Table 1.

**TABLE 2:** Comparison of postoperative complications.

Group	*n*	Flap necrosis (Rate)	Pharyngocutaneous fistula (Rate)	Aspiration (Rate)
Anterolateral thigh flap	3	0 (0%)	1 (33.3%)	1 (33.3%)
Sternohyoid myocutaneous flap	7	1 (14.3%)	1 (14.3%)	1 (14.3%)
*p*		0.168	0.286	0.286

### 3.5 Quality of Life Assessment (QoL Questionnaire)

This study employed a modified Chinese version of the UW‐QOL questionnaire to assess patient QoL preoperatively and 3 months postoperatively. The higher scores indicate better quality of life. For example, Patient 1 had a preoperative total score of 82, with the following domain scores: pain, 10.5; appearance, 9.5; recreation, 9.5; swallowing, 10.5; speech, 10.5; taste, 11.5; saliva, 10.5; and mood, 9.5. At 3 months postoperatively, the total score improved to 86, with domain scores as follows: pain, 11.5; appearance, 8.5; recreation, 9.5; swallowing, 11.5; speech, 11.5; taste, 11.5; saliva, 11.5; and mood, 10.5. As summarized in Table 3, all patients demonstrated improvements in total scores and in most individual domains. The mean total QoL score increased from 80.9 preoperatively to 85.3 at 3 months postoperatively. Improvements were observed across most domains, including Pain (+1.8), Swallowing (+0.9), Mood (+0.9), Recreation (+0.8), Taste (+0.6), and Speech (+0.2), while Saliva (+0.1) showed minimal change and Appearance (−0.9) slightly declined. As shown in Table 4, QoL scores improved 3 months postoperatively for both groups, suggesting that phonation reconstruction improved patients' postoperative quality of life. However, no statistically significant differences were observed between preoperative and postoperative scores (*p* > 0.05).

**TABLE 3:** Patient quality of life scores preoperatively and 3 months postoperatively assessed by the modified Chinese UW‐QOL questionnaire.

Patient no.	Timepoint	Pain	Appearance	Recreation	Swallowing	Speech	Taste	Saliva	Mood	Total score
1	Preoperative	10.5	9.5	9.5	10.5	10.5	11.5	10.5	9.5	82
1	3 months postoperative	11.5	8.5	9.5	11.5	11.5	11.5	11.5	10.5	86
2	Preoperative	8.5	9.5	9.5	10.5	10.5	10.5	11.5	9.5	80
2	3 months postoperative	11.5	8.5	10.5	11.5	10.5	10.5	10.5	10.5	84
3	Preoperative	9.5	9.5	9.5	10.5	10.5	10.5	11.5	9.5	81
3	3 months postoperative	12.5	10.5	11.5	11.5	9.5	11.5	11.5	9.5	88
4	Preoperative	9.5	9.5	8.5	10.5	10.5	10.5	11.5	9.5	80
4	3 months postoperative	11.5	8.5	8.5	10.5	10.5	10.5	11.5	10.5	82
5	Preoperative	9.5	10.5	9.5	10.5	10.5	10.5	10.5	9.5	81
5	3 months postoperative	11.5	8.5	10.5	11.5	10.5	11.5	10.5	10.5	85
6	Preoperative	11.5	9.5	8.5	9.5	10.5	10.5	11.5	10.5	82
6	3 months postoperative	11.5	8.5	9.5	10.5	11.5	11.5	11.5	11.5	86
7	Preoperative	10.5	10.5	8.5	10.5	10.5	10.5	11.5	9.5	82
7	3 months postoperative	11.5	8.5	9.5	11.5	10.5	11.5	11.5	9.5	84
8	Preoperative	9.5	9.5	9.5	10.5	10.5	10.5	10.5	9.5	80
8	3 months postoperative	11.5	8.5	10.5	11.5	11.5	11.5	11.5	10.5	87
9	Preoperative	8.5	9.5	8.5	10.5	10.5	10.5	11.5	9.5	79
9	3 months postoperative	10.5	8.5	9.5	11.5	9.5	11.5	11.5	10.5	83
10	Preoperative	9.5	9.5	9.5	10.5	10.5	11.5	11.5	9.5	82
10	3 months postoperative	11.5	9.5	9.5	11.5	11.5	11.5	11.5	11.5	88

**TABLE 4:** Comparison of quality of life before and 3 months after surgery (Mean ± standard deviation).

Group	*n*	Preoperative	3 months postoperative	Difference
Anterolateral thigh flap	3	81 ± 1	86 ± 2	5 ± 1.732
Sternohyoid myocutaneous flap	7	80.86 ± 1.215	85 ± 2.160	4.143 ± 1.864
*p*		0.361	0.636	0.985

Abbreviation: *n*, number of patients.

As shown in Figure 3B–D, the patient demonstrated good recovery on the first day, 1 week, and 1 month after surgery. After flap survival, a functional airway is established between the tracheal stump and the hypopharyngeal region beneath the tongue base. As a result, phonation can be achieved by manually occluding the tracheostoma with a finger, eliminating the need for external assistive devices such as an electrolarynx.

## 4 Discussion

Preliminary findings suggest that both ALT and sternohyoid myocutaneous flaps are viable options for phonation reconstruction following total laryngectomy. When utilizing the free ALT flap, particular attention should be given to the thickness of the donor site skin. Ideally, the flap should be thin enough to be pinched between two fingers, as excessive subcutaneous tissue may hinder the formation of a tubular structure. Perforator vessels should be identified prior to the flap design, and the flap should be planned according to the precise location of these vessels. To prevent pharyngocutaneous fistula, meticulous suturing of the hypopharyngeal mucosa is essential. By modifying the anastomosis between the phonation tube and the hypopharynx to a purse‐string suture technique, we reduced tissue tension and enhanced anastomotic integrity, resulting in a noticeable decrease in the incidence of pharyngocutaneous fistula compared to that of previous methods. Additionally, based on our clinical experience, an upward‐curved design of the phonation tube appears to reduce the risk of aspiration. However, this observation lacks quantitative validation and warrants further investigation. For the sternohyoid myocutaneous flap, narrower flaps should be considered as they are less prone to necrosis. Preoperative assessment should include consideration of the patient's age and nutritional status. Since the blood supply to this flap was derived from the cutaneous perforators of the infrahyoid branch of the superior thyroid artery, inadequate perfusion warranted the selection of an alternative flap for phonation reconstruction. Despite the considerable number of patients undergoing TL, the methods of voice rehabilitation vary due to regional differences, thereby resulting in small cohorts of patients who underwent the same voice reconstruction method. As such, quantifying the success of various techniques, including phonation success and aspiration rates, becomes challenging. Furthermore, current reconstruction methods focus exclusively on laryngeal phonation without integrating swallowing, breathing, and phonation into a functional whole. Postoperative issues, such as normalizing these functions, remain inadequately addressed.

TL significantly alters self‐perception, social interactions, and societal roles [14]. Addressing quality of life alongside survival rates remains a critical challenge requiring further exploration. The first Greek study evaluating post‐laryngectomy quality of life in patients with cancer revealed overall satisfactory outcomes but highlighted various negative impacts. Large‐scale studies are needed to thoroughly investigate quality of life among patients with laryngeal cancer [15].

Postoperative voice quality is an important marker of successful phonation reconstruction. In our cohort, only modest changes were noted in the speech domain at 3 months, but this should be interpreted with caution. Since most patients retained phonation preoperatively, restoration to a comparable level after tumor resection and flap reconstruction can already be considered a favorable outcome. The primary aim of surgery remains tumor eradication and survival, while functional recovery is a secondary but meaningful objective. Longer follow‐up may reveal further improvements as patients adapt and undergo rehabilitation. Importantly, compared with traditional total laryngectomy, where patients typically lose their natural voice and depend on artificial devices, both ALT and sternohyoid myocutaneous flaps provided acceptable phonation. Similar outcomes have been described in the literature. Patients who underwent ALT phonatory tube reconstruction demonstrated wider fundamental frequency and semitone ranges compared with pneumatic devices, although voice handicap and intelligibility scores were similar [16]. Reconstruction with infrahyoid musculocutaneous flaps produced clear, strong, and stable speech at 1 year after surgery [17]. Our findings are consistent with these reports and support the feasibility of flap‐based phonation reconstruction for functional voice restoration.

The free ALT flap is valued for its large donor area, reliable perforators, and adaptability in creating a phonation tube [16, 18]. It is effective for TL, particularly when combined with hypopharyngeal defect repair and phonation reconstruction. In contrast, the sternohyoid muscle flap, while suitable for phonation reconstruction, is limited in hypopharyngeal repair due to its thinness, restricted mobility, and unreliable vasculature. However, it is easy to harvest and avoids neck bulging or tightness during phonation tube formation. Notably, the microvascular surgical techniques required for voice reconstruction using the free ALT flap are widely applied across multiple specialties, including orthopaedics, plastic surgery, vascular surgery, and oral and maxillofacial surgery. Although the microvascular surgical techniques are less commonly used in otolaryngology, the scope of care within the field of otolaryngology can significantly expand upon skill acquisition. This study involves the use of the free ALT flap to design a voice prosthesis. If otolaryngologists lack experience with free ALT flap surgery, they can collaborate with experts from plastic surgery, oral and maxillofacial surgery, or orthopaedics to perform the procedure.

Risk factors for pharyngocutaneous fistula formation after TL include age, smoking, T stage, prior radiotherapy, and preoperative albumin levels [19]. Fistula incidence increases significantly following radiotherapy [20]; smoking > 20 pack‐years and previous neck radiotherapy are major risk factors. Minimizing these factors can reduce fistula occurrence [21], although consensus on the most accurate risk factors remains absent [22]. The suturing technique at the hypopharyngeal anastomosis is also an important factor contributing to the formation of pharyngocutaneous fistula. When the anastomosis is closed using a mattress suture technique, it reduces incision tension and results in eversion of the wound edges, which helps decrease the incidence of fistula formation. Given that preoperative radiotherapy can complicate surgical procedures and increase the risk of postoperative complications, none of the patients in this study received radiotherapy prior to surgery. Adjuvant radiotherapy may be considered once satisfactory postoperative healing is achieved. In ALT flap harvesting, identifying and preserving perforating arteries and veins with meticulous microvascular anastomosis is essential. Excessive muscle dissection of the sternohyoid flap should be avoided to prevent necrosis. Postoperatively, both groups experienced one pharyngocutaneous fistula case, likely associated with insufficient nutritional support and short‐term surgical site effusion. Enhanced nutrition and wound care resolved these within 15 days.

The length of the sternohyoid flap phonation tube (6–7 cm) reduced the risk of aspiration from solid foods. Severe aspiration might require surgical tube adjustment to decrease its diameter. The curved ‘sickle‐shaped’ configuration of the ALT flap phonation tube helped prevent significant aspiration, even if some saliva or liquid entered the tube. The smaller calibre of the sternohyoid flap phonation tube served as the primary mechanism to reduce the risk of aspiration.

Nutritional status is critical for treatment success, supporting wound healing and restoring physical and mental health. Swallowing difficulties or functional loss often necessitate long‐term enteral nutrition despite intact digestive tract function [23]. Careful perioperative evaluation of the nutritional status of patients is essential. Preoperative nutritional support adjusts indicators to acceptable levels, eliminating contraindications. Timely blood transfusions are essential for patients experiencing significant trauma and blood loss during surgery. Postoperative care requires continuous monitoring of nutritional indicators. When necessary, blood or protein supplementation should be provided alongside intravenous and enteral nutrition to maintain adequate nutritional status. In this study, the nasogastric tube was typically retained for an average duration of 2 weeks. If the surgical site demonstrated good healing, patients could begin practicing oral intake of water, followed by a gradual transition from soft food to semi‐liquid and liquid diets, progressively advancing to a normal diet. If a pharyngeal fistula occurred within the first 2 weeks postoperatively, the nasogastric tube was maintained, and local wound care, nutritional support, and regular dressing changes were performed. The timing for resuming oral intake was adjusted based on the recovery of the fistula. The phonation tube's inner catheter was removed approximately 2 weeks postoperatively, at which point patients could begin phonation training and speech practice.

Phonation reconstruction after TL represents a continuously evolving process that relies on the accumulation of experience and refinement, including patient selection, choice of phonatory tube flap, and surgical proficiency. Owing to the relatively small sample size and short follow‐up duration in our current study, the observed phonation outcomes remained stable during the follow‐up period; however, the long‐term effects warrant further observation and analysis. Although the observed rates of fistula and aspiration in our cohort may appear relatively high, it should be emphasized that the sample size was limited, and a single case could substantially influence the percentages. Previous studies on ALT flap reconstruction have reported a late pharyngocutaneous fistula rate of approximately 5.3% [24], while systematic reviews indicate that the incidence of fistula with ALT flaps generally ranges from 7% to 17%, compared with rates as high as 34% for pectoralis major myocutaneous flaps [25]. With respect to the sternohyoid myocutaneous flap, its application in phonation reconstruction after total laryngectomy is supported by only limited evidence. One study reported that the use of the infrahyoid musculocutaneous flap for voice rehabilitation after total laryngectomy was overall safe and reliable, with no cases of flap necrosis and with most patients regaining fluent and natural speech [26]. Taken together, these data underscore that, when performed by an experienced surgical team, our phonation reconstruction approaches remain within an acceptable safety profile, despite the small sample size and limited statistical power. Observations to date suggested a gradual improvement in the phonatory quality over time, potentially due to increased and smoother airflow following flap absorption and atrophy. However, objective measurement and statistical analysis are necessary to draw scientifically valid conclusions, and further evaluation of phonatory outcomes is warranted. The present data should therefore be interpreted as preliminary findings. With the accumulation of further clinical experience and expansion of the case series, we hope to draw more definitive and persuasive conclusions.

## 5 Conclusions

Preliminary findings suggest that both ALT and sternohyoid myocutaneous flaps are viable options for phonation reconstruction following total laryngectomy. Phonation reconstruction contributed to a moderate improvement in the patients' QoL post‐laryngectomy. The postoperative follow‐up period ranged from 3 – 18 months. Preliminary investigations suggested that myocutaneous flap‐based phonation reconstruction is a viable approach; however, it should not be implemented indiscriminately. Careful assessment of the specific condition of each patient is essential for selecting the most appropriate type of myocutaneous flap. Efforts must also be made to mitigate risk factors, such as flap necrosis, pharyngocutaneous fistula formation, and postoperative coughing. Furthermore, prioritizing perioperative nutritional support, enhanced postoperative management, and timely medical intervention is crucial for improving surgical success rates and achieving better prognostic outcomes.

## Author Contributions

**Hongming Wei:** writing – review and editing, writing – original draft, visualization, validation, methodology, investigation. **Jian Li:** methodology, formal analysis. **Huilei Dong:** visualization, investigation. **Yang Xu:** methodology, investigation. **Xin Li:** data curation, investigation. **Zhu Liu:** software, methodology. **Tie Lu:** visualization, investigation. **Bin Li:** investigation, validation. **Zhendong Li:** resources, supervision, validation. **Hongwei Liu:** conceptualization, resources, project administration, supervision, validation, writing – review and editing.

## Ethics Statement

The studies involving human participants were reviewed and approved by the Medical Ethics Committee of Liaoning Cancer Hospital & Institute.

## Consent

Written informed consent was obtained from all patients for the publication of this study and any accompanying images.

## Conflicts of Interest

The authors declare no conflicts of interest.

## Supporting information


**Data S1:** cam471294‐sup‐0001‐DataS1.docx.

## Data Availability

The data that support the findings of this study are available from the corresponding author upon reasonable request.
