# Supplementary material for: Preliminary Study on Phonation Reconstruction Using Free Anterolateral Thigh and Sternohyoid Myocutaneous Flaps After Total Laryngectomy
Source: Cancer Med. 2025 Oct 8;14(19):e71294. doi: 10.1002/cam4.71294 (PMC12505021; doi:10.1002/cam4.71294)
Supplement: Supplementary file 1 — Data S1: cam471294‐sup‐0001‐DataS1.docx. [file CAM4-14-e71294-s001.docx]

**Washington University Quality of Life Questionnaire - Fourth Edition**
*(Total of 8 items, each with a maximum score of 12.5. with one point deducted for each progressive decline in function.)*

1. **Pain** (Choose one box and check it)
   □ A I have no pain
   □ B I have mild pain that does not require medication.
   □ C I have moderate pain that can be controlled with common painkillers (e.g., aspirin)
   □ D I have severe pain that can only be controlled with prescription painkillers (e.g., morphine)
   □ E I have severe pain that cannot be controlled with any medication
2. **Appearance** (Choose one box and check it)
   □ A My appearance has not changed.
   □ B My appearance has changed slightly.
   □ C I am bothered by my appearance, but I remain active and positive.
   □ D I feel significantly disfigured, and my activities are limited as a result.
   □ E I cannot be around others because of my appearance.
3. **Recreation** (Choose one box and check it)
   □ A I have no restrictions on recreational activities at home or outside.
   □ B There are some things I cannot do, but I can still go out and enjoy recreational activities.
   □ C I often wish I could go out more, but I am unable to do so.
   □ D My recreational activities are very limited; I mostly stay at home watching TV.
   □ E I am unable to engage in any recreational activities.
4. **Swallowing** (Choose one box and check it)
   □ A I can swallow as I did before.
   □ B I cannot swallow certain solid foods.
   □ C I can only swallow liquid foods.
   □ D I cannot swallow because food enters the wrong passage and causes choking.
5. **Speech** (Choose one box and check it)
   □ A I speak as I did before.
   □B I have difficulty with certain words, but people can understand me on the phone.
   □ C Only my family and friends can understand what I say.
   □ D No one can understand what I say.
6. **Taste** (Choose one box and check it)
   □ A I can taste food normally.
   □ B I can taste most foods.
   □ C I can only taste certain foods.
   □ D I cannot taste any food.
7. **Saliva** (Choose one box and check it)
   □ A My saliva is normal.
   □ B I have less saliva than normal, but it is sufficient.
   □ C My saliva is insufficient.
   □ D I have no saliva.
8. **Mood** (Choose one box and check it)
   □ A My mood is good and not affected by my condition.
   □ B My mood is generally good, though occasionally affected by my condition.
   □ C I am neither in a good mood nor depressed due to my condition.
   □ D I feel partially depressed due to my condition.
   □ E I feel very depressed because of my condition.
